# Loneliness, Implicit-Self and Digital Literacy

**DOI:** 10.3389/fpsyg.2022.701856

**Published:** 2022-04-19

**Authors:** Dong Woo Ko, Ji-yeon Lee, Hyesuk Kim

**Affiliations:** ^1^College of HUFS Business School, Hankuk University of Foreign Studies, Seoul, South Korea; ^2^Counseling Psychology, Graduate School of Education, Hankuk University of Foreign Studies, Seoul, South Korea

**Keywords:** loneliness, implicit self, digital literacy, mediated moderation, mindset

## Abstract

Digital literacy is becoming more important because of the skillset of functioning in online is becoming a necessary skill set in daily life. The purpose of this study was to examine the association between loneliness and digital literacy and the mediation effect of motivation in that association. Also, this study examined the moderating effect of mindset in the meditation effect of motivation in the association between loneliness and digital literacy. 287 respondents were recruited from online survey in United States. To investigate the direct effect of loneliness on digital literacy, the mediating effect of motivation, and the moderating of mindset on the mediating effect, this research employed a mediated moderation model. The findings of this research suggest that prevention-focused motivation mediated the effect of loneliness on digital literacy and the effect was moderated by a fixed mindset (as opposed to a growth mindset). The result of the study contributes to the literature by examining how loneliness could impede acquiring digital literacy through prevention-focused motivation and fixed mindset.

## Introduction

People have a fundamental desire to maintain a certain quality of relationship with others, and Baumeister and Leary (1995) discussed how that desire is important in many aspects of our daily lives. Similarly, when there is a discrepancy between the desired quality of a social relationship and an actual relationship, people naturally feel negative; this is defined as loneliness (Peplau and Perlman, 1982). One interesting thing is, according to a recent report, more than 60% of the United States population has been a victim of loneliness, and this percentage has been continuously rising (CIGNA, 2020). Why do so many people experience the negative feelings these days? Various social and environmental factors could influence the cause of the phenomena, and the development of the Internet is considered as one of them. In fact, there has for long been discussion to understand the causal relationship between loneliness and internet usage; this is the Internet paradox, as to whether the Internet increases psychological well-being or decreases it (Kim et al., 2009). Regarding the issue, a large segment of the research has reported that the development of the Internet and social media is one of the important factors exacerbating loneliness (Caplan, 2007; Sum et al., 2008; Nowland et al., 2017; Savolainen et al., 2020; Masur, 2021) in that the internet reduces the chance of the communicators meeting face to face and make the conversation superficial and limits them from expressing their feelings (Ceyhan and Ceyhan, 2008; Bian and Leung, 2014). On the other hand, there is a research stream discussing the positive influences of the Internet on consumers’ psychological well-being (Shaw and Gant, 2002) by enabling communication between people separated by great distances. Even though there are conflicting views regarding the Internet paradox being a causal issue, the literature commonly suggests the important relationship between loneliness and the Internet usage (Masur, 2021). Therefore, in this study, we explored how loneliness is associated with how people operate in the digital world by focusing on the concept of digital literacy. Digital literacy is the ability to understand instructions from visual cues and operate a digital device with an understanding of the rules of the digital environment. This requires complex cognitive, motor, sociological, and emotional skills (Eshet, 2004). Therefore, lack of digital literacy keeps individuals from performing to their full potential as a competent student, an employee, or a well-functioning citizen (Meyers et al., 2013), as the world becomes digitalized. To operate in the digital world with competence, one needs to be openminded about learning new skill sets. In this, one might be affected by the need for motivation to learn and improve one’s digital skills despite the risk involved in the use of digital media. To investigate the underlying mechanism of the effect of loneliness on digital literacy, this research employed the concept of motivation and mindset and investigated how loneliness influences individuals’ motivation and mindset, and the effect of their interaction on digital literacy. This study contributes to understanding the mechanisms of loneliness and digital literacy so that lonely people in a digitalized society can adapt to the changes well without falling behind. The rest of this study is organized as follows. First, based on past literature related to loneliness and digital literacy, we will suggest the relationship and understand the underlying mechanism between loneliness and digital literacy. Then, we will analyze the same through a two-step analysis. Finally, the academic and empirical contributions of this study will be discussed.

### Loneliness, Motivation, and Mindset

Loneliness is an unpleasant experience that is derives from deficiencies in social relationships (Russell et al., 1980; DiTommaso and Spinner, 1997). According to a recent study, digital literacy education can be used to reduce social loneliness (McGinty, 2020). However, in this study, we focus on the relationship between loneliness and digital literacy based on the motivations that lonely people might have. Research indicates that loneliness is associated with learning motivation (Wang, 1989) as well as learning ability (Zhang et al., 2007). The relationship between loneliness and low learning ability is usually discussed based on burnout caused by academic pressure or homework overload (Balogun et al., 1996; Lingard et al., 2007; Zhang et al., 2007). Also, this tendency is not limited to students. Even in organizational structure, the difficulties that people face in learning and being creative in the workplace are reported to be caused by loneliness and psychological fatigue (Ozcelik and Barsade, 2018). Considering the emotional fatigue caused by loneliness, which ultimately interferes with the learning process, it is possible to predict negative relationships between loneliness and digital literacy, as learning to operate in the digital world requires constant effort to understand new technologies and carefully select appropriate new technologies.

To understand how loneliness affects digital literacy, it is important to understand how loneliness is associated with motivation (i.e., prevention vs. promotion focus). According to a study by Lee et al. (2019), the lonelier a person is, the more addicted they are to online games, and the cause is explained through motivation. Lonely people feel more to be at risk than those who are not lonely people, and their perception of risk is reflected in their tendency to base their actions on prevention-focused motivations. Regulatory focus theory understands people’s behaviors by the two distinct strategies they follow in making decisions (Higgins, 1997). Even for those who pursue the same goals, some people focus on the “gain” aspect, that is, the gain to be made from accomplishing the goal. Some people, on the other hand, focus on the “non-loss” aspects, that is, the loss that would be prevented by achieving the goal. The two types of people base their strategies to achieve the same goals on these two distinct focuses. Thus, to achieve the same goal, the motivations are different and they affect people’s behavior (Higgins, 1997). The motivation that comes from proactive thoughts that are focused on the gain is called promotion-focused motivation, while the one arising from thoughts focused on security concern or safety is called prevention-focused motivation (Higgins, 1997). If the actual social relationships fall short of the desired social relationships the gap makes people feel lonely. Loneliness activates the self-defensive mechanism that becomes manifest as prevention-focused motivation. Also, mindset must be considered as one of the things that influence motivation (e.g., Rhew and Piro, 2018), which, in turn, affects people’s strategic approach in dealing with loneliness.

### Growth and Fixed Mindset

According to mindset research on personality, self-concept, and intelligence change depending on how and with what mindset people treat themselves (Dweck et al., 1995). The mindsets here refer to lay belief that people can malleably develop personal qualities and traits (Dweck et al., 1995). Chiu et al. (1997), for example, distinctly identify the mindsets in two ways: fixed mindset and growth mindset. Individuals who have fixed mindset believe that their personal traits, such as moral character or personality, are fixed so that they cannot develop or change their personal traits through their endeavor. On the other hand, people who have a growth mindset believe that they can develop or modify their personal attributes if they try hard enough. People possess both the mindsets, fixed mindset, and growth mindset, at the same time. One of the mindsets comes into play when particular environmental factors exist and the mindset affects a particular part of the action. The mindset, as a part of human character, has been explored psychologically in academic and sociological fields. Recently, research has been conducted on mindset as a part of consumer behavior as well (e.g., Park and John, 2010; Yorkston et al., 2010; Kwon et al., 2016; Seo et al., 2021). Rai and Lin (2019) demonstrated that the mindset affects customers’ financial decision making according to their belief in personality traits. They suggested that people who have a growth mindset are likely to focus on promotion motivation and be dedicated to positive results whereas people with a fixed mindset are likely to be focused prevention-motivation, and be dedicated to negative results. Here we need to consider that the feeling of loneliness will influence their beliefs about their personal qualities. There is a basic motivation called *status quo* that drives people to try to return to a stable state (Samuelson and Zeckhauser, 1988). In other words, the negative experiences of their inability to change their personal traits despite the efforts would make their fixed mindset salient. Also, the prevention-focused motivation that is caused by loneliness will mediate the activation of a fixed mindset. Therefore, our first hypotheses:

H1. The lonelier people feel, more likely they are to have a fixed mindset.

H2. Prevention-focused motivation will mediate the effect of loneliness on a fixed mindset.

### Digital Literacy and Loneliness

As digitalization progresses, social, cognitive, and technical abilities, are becoming essential for solving various problems that arise in the digital environment. This special knowledge and ability are called digital literacy (Lenham, 1995; Papert, 1996; Inoue et al., 1997; Eshet and Amichai-Hamburger, 2004; Reddy et al., 2020). Having digital literacy is, thus, acquiring a large, varied, and complex skillset necessary to function effectively in digital environments (Eshet and Amichai-Hamburger, 2004). More specifically, digital literacy is conceptualized as including the ability to read visual instructions, utilize digital skills to reproduce meaningful messages to meet specific needs, evaluate the quality of digital information (e.g., careful selection of relevant information from the retrieved information), and understanding the cyberspace “rules” and following them in framing the communications (Eshet and Amichai-Hamburger, 2004; Reddy et al., 2021). Especially since the terrifying and highly infectious virus, COVID-19 has accelerated the transformation of the world into a digital-world (e.g., Priyono et al., 2020; Trenerry et al., 2021), it is important to possess digital literacy. Although people are expected to keep the physical distance to prevent the spread of infection, yet they are expected to continue their daily lives. Now the ability to effectively understand and use the various functions in the digital world does not remain an option but has become a skillset for performing ordinary tasks such as ordering food or paying for parking without having human contact, which is necessary for survival.

Experiences of loneliness usually involve negative feelings, such as anxiety, dissatisfaction, depression, and pessimism (Weiss, 1973). There have been several empirical studies regarding the association between loneliness and problems with the use of the internet such as compulsive internet use (Savolainen et al., 2020), SNS addiction (Reid and Reid, 2007), and online gaming addiction (Lee et al., 2019). In particular, according to a recent research, smartphone addiction among adolescents appears to have reached serious levels, and loneliness or self-regulation is seen as the cause of these negative outcomes (Lãzãroiu et al., 2020; Lewis et al., 2020; Porter et al., 2020; Scott et al., 2020; Taylor et al., 2020). Mahapatra (2019) suggested that adolescents feel lonely based on poor academic performance or family strife, which is slightly different from the causes of the loneliness felt by other generations. However, as various studies have shown, it is important to examine how loneliness would impede or accelerate the adaptive use of the internet (i.e., digital literacy), which has not been empirically investigated before.

The process of acquiring digital literacy requires the ability to learn new things such as a high-level cognition, which can be complicated by the continuous development of digital technology (Eshet, 2012). However, people who are psychologically and emotionally unstable due to loneliness show a tendency to avoid risks rather than accepting digital world as an essential part of being progressive (Lee et al., 2019). Although there are no studies that directly show the relationship between loneliness and digital literacy, previous studies in education confirmed that loneliness impedes learning because it acts as an emotional barrier (Kubey et al., 2001). Long-term loneliness linked to not only physical and mental health, but it also significantly influences learning achievements (Wang, 1989), dropout rates in higher education (Medora and Woodward, 1986; Cacioppo et al., 2006), and learning burnout (Lin and Huang, 2012). Therefore, loneliness is expected to cause a disinclination in learning the new digital technology which requires constant updating to remain competent in the fast-changing digital world. The prevention-focused motivation caused by loneliness will cause a behavior aimed at maintaining the *status quo* rather than taking the risk of learning something new that calls for a high level of cognitive process, and this is a cause for lower digital literacy. Also, the fixed mindset caused by loneliness will make people believe that they cannot change even if they try. This mindset will lower the motivation to learn new things. The fixed mindset, therefore, will influence digital literacy by moderating loneliness and prevention-focused motivation. Therefore, we hypothesize that:

H3. The people who feel lonelier have lower levels of digital literacy.

H4. The prevention-focused motivation will mediate the effect of loneliness on digital literacy.

H5. The people with a fixed mindset are more likely to have a lower level of digital literacy.

H6. The fixed mindset will moderate the effect of loneliness on digital literacy.

H7. The fixed mindset will moderate the indirect effect of loneliness on digital literacy through a prevention-focused mindset.

## Materials and Methods

### Data and Sample

To investigate the proposed hypotheses, we collected data from online panels in the United States. The survey was posted in Amazon Mechanical Turk and recruited 290 participants. Because seven participants did not finish the survey, 283 responses were collected (Male: 54.4%). The average response time was approximately 9 ∼ 10 mins, and please see Table 1 for the demographics of participants.

**TABLE 1:** Demographic information.

Subject		Frequency (percentage)
Age	19–29	70 (24.7%)
	30–39	101 (35.7%)
	40–49	54 (19.1%)
	50–59	39 (13.8%)
	60–69	16 (5.7%)
	Over 70	3 (1.1%)
Race	White/caucasian	214 (75.6)
	African American	26 (9.2%)
	Hispanic	19 (6.7%)
	Asian	22 (7.8%)
	Others	2 (0.7%)
Education	Not completed high school	1 (0.4)
	High school graduate or college	66 (23.3)
	College graduate (4 years)	150 (53%)
	Postgraduate degree	66 (23.3)
Income	∼ $30,000	47 (16.6%)
	$30,001 ∼ $60,000	108 (38.2)
	$60,001 ∼ $90,000	66 (23.3%)
	$90,001 ∼ $120,000	36 (12.7%)
	$120,001 ∼	26 (9.2%)

As participants visited the survey website, they learned that they were going to be asked several short questions designed for efficiency. If respondents agreed to participate in the study, they were to begin the survey by answering demographic questions. As participants progressed with the questionnaire, they found questions on loneliness, digital literacy, regulatory focus, and mindset. To measure loneliness, the UCLA scale refined by Russell et al. (1978) was used. This section contained 20 questions, and participants were asked to check one out of four answers that described them best. The answers ranged from “I often feel this way” to “I never feel this way.” That is, as the number increases, the loneliness decreases. It is, however, reverse coded for the analysis. Thus, as the loneliness increases the number increases in the analysis (Cronbach alpha = 0.965). After participants completed the loneliness questions, they were asked to answer the scales for digital literacy. This was measured through the scale proposed by Ng (2012). This section was composed of 11 questions in three subcategories; seven questions were related to the technical dimension, two questions were related to the cognitive dimension, and two questions were related to social-emotional dimensions. The participants responded on a 5-point Likert scale in which 1 indicated a low level of digital literacy and 5 indicated a high level of digital literacy. Because the goal of this research was to understand the overall digital literacy rather than to measure the subcategories, the average of 11 questions was used in the analysis (Cronbach alpha = 0.852). Regarding the motivation, the regulatory focus scale, proposed by Higgins et al. (2001) was used. Eleven questions were divided into two subcategories –six questions for measuring promotion-focused motivation and five questions for measuring prevention-focused motivation. The participants responded on a five-point Likert scale. As Higgins et al. (2001) suggested we combined the two subcategories by subtracting prevention-focused motivation from promotion-focused motivation (Cronbach alpha for promotion-focused motivation = 0.849; Cronbach alpha for prevention-focused motivation = 0.903). Thus, as the number increases, it indicates a higher level of promotion-focused motivation. Lastly, we investigated the respondents’ mindset (fixed mindset vs. growth mindset), the measure of the implicit-self-theory scale was used. This scale, suggested by Levy et al. (1998), consisted of eight questions. The first four questions were designed to measure incremental theories (growth mindset) and the remaining four questions were to measure the fixed mindset (entity theorists), the participants responded to the questions on a 5-points Likert scale. Because the concepts of growth mindset and fixed mindset are contradictory, when the growth mindset is low, for example, it also means that the fixed mindset is high and vice versa. Thus, growth mindset questions were recoded into fixed mindset. The lower number indicated a higher level of growth mindset, and as the number increased, it indicated the higher level of fixed mindset (Cronbach alpha = 0.92). The analysis consisted of two steps. First, to understand the characteristics of loneliness, we investigated the effect of loneliness on mindset and its relationship with motivation. Second, based on the characteristics of loneliness, this research conducted a moderated mediation analysis to understand the relationship between loneliness and digital literacy, and the moderated roles of mindset in the model. We employed mediation analysis and moderated mediation analysis to investigate the proposed research questions and conceptual model. In particular, the collected data was based on continuous variables, not group variables. Thus, the study was based on PROCESS, the most suitable process for these data.

### Loneliness and Mindset

**FIGURE 1:**
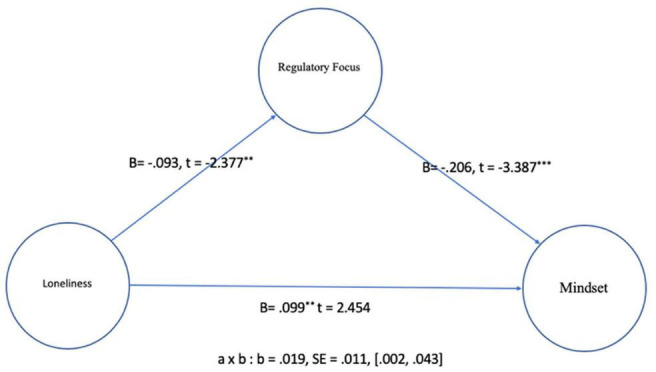
Mediation effect of regulatory focus. **p < 0.01, ***p < 0.001.

To investigate the relationship between loneliness, regulatory focus, and mindset (H1, H2, and H3), the mediation test was conducted (Hayes, 2013; PROCESS macro Model 4). The model facilitates investigation of whether regulatory focus mediates the effect of loneliness on mindset. The analysis uses 5000 bootstrapping method to instigate the indirect effect of regulatory focus on mindset while gender, age, race, and education level were controlled (see Figure 1). Results show that there is the direct effect of loneliness on mindset (*b* = 0.099, SE = 0.040, *p* < 0.05). The positive coefficient (b) indicates that as one unit of loneliness increases, people are more likely to have a fixed mindset, up to 0.099.

Also, the results indicated that there was a negative relationship between loneliness, regulatory focus, and mindset. Specifically, as people feel lonelier, they are more likely to have the prevention-focused motivation (*b* = −0.093, SE = 0.039, *p* < 0.05) and regulatory focus negatively influenced fixed mindset. That is, as the promotion-focused motivation increased, growth mindset became more salient (*b* = −0.206, SE = −0.061, *p* < 0.01). Regarding indirect effect, the results of bootstrapping analysis showed that the regulatory focus significantly influenced the effect of loneliness on mindset [*b* = 0.19, SE = 0.011, (0.002,0.043)]. The results of mediation test supported the hypotheses 1, 2, and 3. As people feel lonelier, they are more likely to have a fixed mindset and the prevention-focused motivation mediates the effect of loneliness on the salience of their fixed mindset. The results show that as people feel loneliness, they are more likely to have a fixed mindset, which means that they believe they cannot change themselves no matter how hard they try. In addition, the mediation test showed that the lonelier the people are, the more they are motivated to defend their current state rather than pursue new things, which causes the fixed mindset.

### Loneliness and Digital Literacy

**FIGURE 2:**
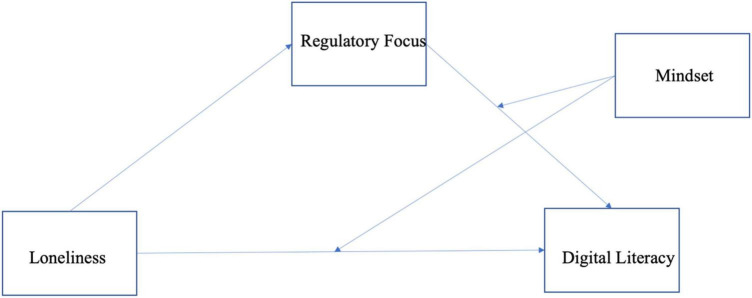
Moderated mediation effect on digital literacy.

How, then, the loneliness influenced the attitude toward digital literacy? This research proposed that the feeling of loneliness would influence the level of digital literacy because lonely people have prevention-focused motivation and fixed mindset. To investigate the relationship, this research employed moderated mediation analysis (Hayes PROCESS macro model 15). The model allowed investigation into whether the different types of mindset (growth mindset vs. fixed mindset) moderated the proposed direct and indirect effect of loneliness on digital literacy that was mediated by motivation. Accordingly, we followed the moderated mediation model with bootstrapping analysis to investigate the indirect effect of motivation (m) on the direct effect of loneliness (x) on digital literacy (y) at various levels of mindset (z) (see Figure 2). The results of the analysis showed that as people feel lonelier, they were likely to show a low level of digital literacy (*b* = −0.670, SE = 0.039, *p* < 0.05) (see Table 2). Direct effect of motivation on digital literacy was positively significant (*b* = 2.064, SE = 0.418, *p* < 0.001) while the effect of mindset on digital literacy was not significant (*b* = −0.215, SE = 0.405, *p* > 1). Also, the model summary indicated that there is a negative but significant effect of loneliness on the motivation (*b* = −0.093, SE = 0.039, *p* < 0.05).

**TABLE 2:** Moderated mediation analysis.

Model	B	SE	*t*	*p*
**Mediator variable: regulatory focus**				
Constant	–0.073	0.428	–0.171	0.864
Loneliness	–0.093	0.039	–2.377	0.018
Gender	0.045	0.113	0.400	0.689
Age	0.007	0.047	0.158	0.874
Race	–0.092	0.059	–1.554	0.121
Education	0.032	0.081	0.400	0.690
**Dependent variable: digital literacy**				
Constant	49.997	2.918	17.134	0.000
Loneliness	–0.697	0.273	–2.552	0.011**
Regulatory focus	2.064	0.418	4.934	0.000**
Mindset	–0.215	0.405	–0.532	0.595
Loneliness × Mindset	–0.204	0.251	–0.811	0.418
Regulatory focus × Mindset	0.891	0.371	2.40	0.017**
Gender	–1.208	0.771	–1.566	0.118
Age	–1.133	0.319	–3.487	0.001
Race	–0.222	0.403	–0.550	0.583
Education	0.337	0.553	0.609	0.543

N = 283. Regulatory focus (the mediator) is centered in the analyses; bootstrap sample = 5,000 B = coefficient SE = standard error. SE = standard Error, **p < 0.01.

The model summary in Table 3 and the analysis of conditional indirect effect analysis (Table 4) consistently show the significant interaction effect between regulatory focus and mindset (*b* = 0.891, SE = 0.371, *p* < 0.05). As shown in the interaction effect between mindset and regulatory focus in the model summary, the conditional effects of the focal predictor (Table 3) suggest that the people who have fixed mindset and promotion-focused motivation will have a high level of digital literacy (growth mindset: *b* = 1.141, SE = 0.527, *t* = 2.163, *p* < 0.05 vs. Fixed mindset: *b* = 2.840, SE = 0.572, *t* = 4.959, *p* < 0.001). Regarding the indirect effect analysis, the results show that the indirect effect is significantly conditional on the level of the mindset (bootstrap lower bound = −0.221, upper bound = −0.002). The indirect effect is not significant on the growth mindset (bootstrap lower bound = −0.028, upper bound = 0.002) while the effect was significant on a fixed mindset (bootstrap lower bound = −0.392, upper bound = −0.044). The interaction effect between loneliness and mindset is not significant (*b* = −0.204, SE = 0.251, *P* > 1). In other words, the results indicated that the indirect effect of loneliness through the motivation on digital literacy is significant when the subject had a fixed mindset. However, the direct effect of loneliness on digital literacy is not moderated by mindset.

**TABLE 3:** Conditional effect of the regulatory focus at values of mindset.

Implicit-self theory	Effect	SE	*t*	*p*
Growth mindset	1.141	0.527	2.163	0.031**
Between	1.990	0.419	4.749	0.001***
Fixed mindset	2.840	0.573	4.959	0.001***

SE = standard Error, **p < 0.01, ***p < 0.001.

**TABLE 4:** Conditional indirect effect (s) of X on Y at Values of the Moderator.

Mid-set	Effect	SE	LLCI	ULCI
Growth mindset	–0.113	0.048	–0.0278	0.002
Between	–0.193	0.093	–0.392	–0.033
Fixed Mindset	–0.273	0.502	–0.392	–0.044

In sum, a two-step analysis was used to characterize lonely people and reveal their impact on digital literacy. The results showed that lonely people had a fixed mindset and prevention-focused motivation. It can be inferred that lonely people are less inclined to pursue new things because of the prevention-focused motivation and fixed mindset, believing that they cannot change themselves with effort. This trend was confirmed once more through the relationship between lonely people and digital literacy. Prevention-focused motivation negatively affected digital literacy. This trend was moderated as the fixed mindset increased.

## Discussion

The purpose of this study was to examine the association between loneliness and digital literacy, and the mediating effect of motivation in that association using a two-step analysis method testing the moderated mediation model. This study examined the moderating effect of mindset in the mediating effect of motivation in the association between loneliness and digital literacy. The results of the study confirmed the association between loneliness and digital literacy, as well as the mediating effect of motivation in the association between loneliness and digital literacy, along with the moderating effect of mindset. The results showed that lonely people are likely to have a fixed mindset and prevention-focused motivation. In other words, lonely people are less inclined to pursue digital literacy because of their prevention-focused motive and fixed mindset, believing that they cannot change themselves with effort. One of the important contributions of this study is that factors derived from loneliness to digital literacy were taken together through a mediated moderation model. Compared to the emotional aspect, motivation and mindset are factors that are difficult to be noticed by others. In this study, we followed an in-depth approach to study the effects of loneliness by understanding the complex internal processes of motivation and mindset. First, the mediation analysis suggests that the self-defensive mechanism stimulated by loneliness plays a key role in mediating the mindset and digital literacy. Second, for people who are more inclined toward a fixed mindset, the mindset moderates the negative indirect effect of loneliness on digital literacy as opposed to people who have a growth mindset. Through the process of showing the relationships between factors influencing digital literacy, it is possible to strategically develop ways to increase digital literacy.

### Theoretical/Managerial Contribution

The findings of this research suggest that prevention-focused motivation mediated the effect of loneliness on digital literacy and that this effect was moderated by a fixed mindset (as opposed to a growth mindset). First, this research fills the existing gaps between loneliness and digital literacy. The results of this study, particularly through its revelation of the internal mechanism of the effect of loneliness on digital literacy through motivation and mindset, would be useful in understanding why some people have more difficulty in adapting to the digital world. Secondly, this study contributes to the mindset literature by extending the scope of the research. The concepts of growth mindset and fixed mindset have been widely used in the field of marketing and education for their role in moderating certain phenomena rather than showing how the mindset is derived. For example, they have been considered as a way to increase brand attitude through advertising slogans (Kwon et al., 2016; Seo et al., 2021). This study, however, shows how the mindset is directly and indirectly affected by loneliness and motivation. It may suggest the importance of adapting an integrative approach in understanding digital literacy by taking the emotional (i.e., loneliness) and cognitive aspects (i.e., mindset) together. Lastly, this study found how loneliness could be an emotional barrier for the effective functioning of an individual in the digital world. Considering that loneliness is an emotional reaction for unmet needs for an emotional connection with others, the results of the study imply that loneliness could be an emotional barrier for learning the skills necessary to function in the digital world by taking prevention-focused motivation. More importantly, the effects of loneliness on learning have a complex structure; loneliness affects motivation (i.e., inclination to be prevention-focused), and the prevention-focused motivation impedes lonely people from having a growth mindset in learning the skills that are necessary to function in the digital world.

Digital literacy is an essential element of life that cannot be avoided. Many experts predict that in the future, we will face a digital world that will be replete with myriad technologies that would require high-level cognitive processes and motivation to learn new skill sets (Butler, 2020; He and Harris, 2020; Keesara et al., 2020). By considering motivation and mindset together with loneliness, this study provides ideas for intervention. For example, to assist people who face difficulty in understanding visual cues and rules of the digital world, the results of this study can be used to help them by exploring their emotional and cognitive barriers to learning how to handle new technologies, and providing them motivational or emotional support. The results of this study also indicate that it would be effective in helping lonely people have promotion-focused motivation and a growth mindset toward increasing their digital literacy. Regarding the intervention, many existing studies in psychology and business have shown that such artificial manipulation in motivation or mindset leads to behavioral change. For example, goal-oriented messages can increase promotion-focused motivation (Keller, 2006; Yeo and Park, 2006), and choosing words that can create faith in growth can influence consumers to have a growth mindset (Kwon et al., 2016; Seo et al., 2021). Therefore, using various motivational and cognitive approaches, along with psychological support, to lessen loneliness would play a key role in helping people acquire digital illiteracy.

### Limitation

Despite the significant contributions to the literature on loneliness and digital literacy, this study has limitations that need to be addressed. First, this study did not directly measure other emotional factors related to loneliness such as feelings of loss or sadness. Because loneliness is an emotional reaction to unmet relational needs, there should be room for sufficient consideration of the relationship between other emotional factors and motivation (or mindset). More specifically, it would be interesting to investigate the effect of the feeling of loss on motivation and mindset in future studies. Second, this study did not investigated ways of improving digital literacy. As previously discussed, the purpose of this study was to accurately understand the complex internal processes of loneliness and digital literacy. Therefore, it has a limitation that it did not exactly tested how to increase digital literacy. In the future study, it would be important to include experiments to verify whether manipulation of motivation and mediation can increase digital literacy.

## Data Availability Statement

The raw data supporting the conclusions of this article will be made available by the authors, without undue reservation.

## Ethics Statement

The studies involving human participants were reviewed and approved by the Hankuk University of Foreign Studies IRB. The patients/participants provided their written informed consent to participate in this study.

## Author Contributions

DK, J-YL, and HK conceptualized and wrote the manuscript. All authors contributed to the article and approved the submitted version.

## Conflict of Interest

The authors declare that the research was conducted in the absence of any commercial or financial relationships that could be construed as a potential conflict of interest.

## Publisher’s Note

All claims expressed in this article are solely those of the authors and do not necessarily represent those of their affiliated organizations, or those of the publisher, the editors and the reviewers. Any product that may be evaluated in this article, or claim that may be made by its manufacturer, is not guaranteed or endorsed by the publisher.
